# Integrative systems‐level analysis reveals a contextual crosstalk between hypoxia and global metabolism in human breast tumors

**DOI:** 10.1002/1878-0261.13762

**Published:** 2024-12-27

**Authors:** Raefa Abou Khouzam, Salem Chouaib, Mohammad Askandar Iqbal

**Affiliations:** ^1^ Thumbay Research Institute for Precision Medicine Gulf Medical University Ajman United Arab Emirates; ^2^ College of Medicine Gulf Medical University Ajman United Arab Emirates; ^3^ INSERM UMR 1186, Gustave Roussy, EPHE, Faculty of Medicine University of Paris‐Saclay Villejuif France

**Keywords:** breast cancer, cancer metabolism, hypoxia, systems biology, Warburg effect

## Abstract

Hypoxia is known to induce reprogramming of glucose metabolism in cancer. However, the impact of hypoxia on global metabolism remains poorly understood. Here, using the systems approach, we evaluated the potential crosstalk between hypoxia and global metabolism using data from > 2000 breast tumors. Tumor samples were scored for hypoxia and 90 metabolic pathways, and these metrics were subjected to an analysis pipeline. Hypoxia showed a very strong association with metabolic aggression and an overall contextual relationship with metabolism. Out of three (M1, M2, and M3) metabolic types in breast cancer, M3 exhibited the strongest relationship with hypoxia; that is, high hypoxic tumors were also metabolically deregulated. Further, the overall correlation pattern between hypoxia and metabolic pathway scores was specific to each type, with M1 showing maximal sensitivity to hypoxia, followed by M2 and then M3. Experimental validation using metabolic inhibitors on cell lines with high or low hypoxia scores further confirmed the metabolic type‐dependence of hypoxia. In addition, evaluation of the impact of hypoxia on cancer pathways other than metabolic ones revealed a potential role of hypoxia in immune evasive characteristic of M3 tumors. Overall, the results suggest a complex interplay between hypoxia and metabolism in the context of human breast tumors, with potential implications for both basic cancer biology and breast cancer therapy.

## Graphical abstract

Breast tumor samples scored for metabolic deregulation (M1 to M3) were given a hypoxia score (HS). The highest HS occurred in patients with strongest metabolic deregulation (M3), supporting tumor aggressiveness. HS correlated with the highest number of metabolic pathways in M1. This suggests hypoxia to be an early event in metabolic deregulation. The overall crosstalk between hypoxia and metabolism was found to be contextual.

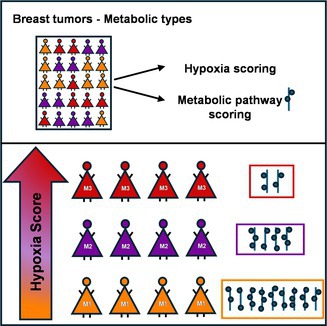

**Abbreviations**
ACOT7: acyl‐CoA thioesterase 7
ADM: adrenomedullin
AKT: protein kinase B
ALDOA: aldolase, fructose‐bisphosphate A
ATP: adenosine triphosphate
BC: breast cancer
BRCA: breast invasive carcinoma
CDKN3: cyclin‐dependent kinase inhibitor 3
DDIT4: DNA damage‐inducible transcript 4
DMEM: Dulbecco's modified Eagle medium
DMSO: dimethyl sulfoxide
DSS: disease‐specific survival
EGA: European Genome‐phenome Archive
ENO1: enolase 1
FBS: fetal bovine serum
HIF: hypoxia‐inducible factor
HS: hypoxia score
LDHA: lactate dehydrogenase A
METABRIC: Molecular Taxonomy of Breast Cancer International Consortium
MIF: macrophage migration inhibitory factor
MRPS17: mitochondrial ribosomal protein S17
mTOR: mammalian target of rapamycin
MXI1: MAX Interactor 1
NDRG1: N‐Myc downstream regulated 1
OS: overall survival
P4HA1: prolyl 4‐hydroxylase subunit alpha 1
PET: positron emission tomography
PGAM1: phosphoglycerate mutase 1
PGK1: phosphoglycerate Kinase 1
PH: proportional‐hazards
PI3K: phosphoinositide 3‐kinase
RFS: recurrence‐free survival
SLC2A1: Solute Carrier Family 2 Member 1
TCGA: The Cancer Genome Atlas
TPI1: triosephosphate isomerase 1
TUBB6: tubulin beta 6 class V
UCSC: University of California, Santa Cruz
VEGFA: vascular endothelial growth factor A
2DG: 2‐deoxyglucose

## 1 Introduction

Hypoxia is a hallmark of solid tumors which arises due to an inadequate supply of oxygen caused by a combination of structural and functional abnormalities in the tumor vasculature [1]. Solid tumors often outgrow their blood supply, leading to regions of inadequate perfusion and oxygen delivery [2]. As a result, pockets of sub‐physiologic oxygen levels ranging from < 5–10 mm Hg exist in tumors [2]. The irregular and tortuous nature of tumor blood vessels, coupled with increased interstitial pressure, vessel leakage, uneven blood flow, and intermittent perfusion hampers efficient oxygen transport to tumor cells and create a heterogeneous distribution of oxygen within the tumor, with some areas experiencing chronic hypoxia while others undergo cyclic fluctuations in oxygen tension [3]. In addition, rapidly proliferating cancer cells consume oxygen at a high rate to meet their metabolic demands for energy production, biosynthesis, and redox balance [4]. This elevated oxygen consumption exacerbates oxygen depletion in the tumor microenvironment, particularly in regions distant from functional blood vessels [5]. The tumor microenvironment, comprising cancer‐associated fibroblasts, immune cells, endothelial cells, cytokines, and extracellular matrix component can further exacerbate hypoxia through paracrine signaling and physical barriers that impede oxygen diffusion [6].

Induction of hypoxia triggers the stabilization and activation of hypoxia‐inducible factors (HIFs), transcription factors that regulate the expression of genes involved in angiogenesis, glycolysis, pH regulation, and other adaptive responses to low oxygen tension [4]. HIF activation plays a central role in orchestrating the cellular and molecular adaptations to hypoxia in solid tumors. The presence of hypoxia in solid tumors has profound implications for tumor progression, metastasis, immune escape, and therapeutic resistance [7, 8, 9]. Accordingly, several studies, including ours, have identified and validated hypoxia signatures in different tumors [10, 11, 12]. The clinical relevance of the hypoxia signature in solid tumors lies in its potential as a prognostic marker, predictive biomarker, and therapeutic target [13]. Understanding the hypoxia signature can provide valuable insights into tumor biology, treatment response, precision medicine, and patient outcomes. Overall, the hypoxia signature holds promise as a clinically relevant indicator that could be exploited for improved patient management.

Metabolic reprogramming in cancer refers to alterations in cellular metabolism that enable cancer cells to sustain their uncontrolled growth and proliferation [14]. Normal cells rely primarily on oxidative phosphorylation, a process that generates energy in the form of ATP through the consumption of oxygen. However, cancer cells often switch to a different metabolic strategy known as aerobic glycolysis, or the ‘Warburg effect’, where they preferentially metabolize glucose to lactate even in the presence of oxygen [15]. This metabolic shift provides several advantages to cancer cells like rapid ATP production, biomolecular synthesis, gene regulation, epigenetic programming, immune escape, redox balance, and adaptation to hypoxia [16, 17, 18, 19, 20]. Besides, metabolic rewiring in cancer has been implicated in chemo‐and radio resistance [21, 22]. Targeting metabolic vulnerabilities in cancer cells, such as inhibitors of glycolysis or glutaminolysis, holds promise as a therapeutic strategy to selectively kill cancer cells while sparing normal cells [23]. Moreover, metabolic imaging techniques like positron emission tomography (PET) can detect changes in tumor metabolism and help guide treatment decisions in cancer patients [24].

Cancer metabolism and hypoxia are intimately interconnected processes with each influencing and exacerbating the effects of the other in solid tumors [25]. Importantly, hypoxia alters cancer metabolism profoundly through a variety of mechanisms [26]. For instance, hypoxia promotes the upregulation of glycolytic enzymes and glucose transporters, leading to increased glucose uptake and lactate production by cancer cells, even in the presence of oxygen (the Warburg effect) [27]. The shift from oxidative phosphorylation to glycolysis allows cancer cells to produce ATP more rapidly under hypoxic conditions [28]. Moreover, hypoxic cancer cells are also able to metabolize alternate carbon source (other than glucose) [29]. The accumulation of lactate in the hypoxic tumor microenvironment, because of Warburg effect, exacerbates acidosis leading to metabolic stress, cellular damage, and immune escape [30, 31, 32, 33]. Moreover, acidic conditions (lactate) promote the release of pro‐angiogenic factors and proteases that facilitate tumor progression and metastasis [34, 35]. Therefore, understanding the complex interplay between cancer metabolism and hypoxia is critical for developing potential innovative therapeutic strategies that target metabolic vulnerabilities and exploit the hypoxic microenvironment to improve treatment outcomes for cancer patients. Therapeutic approaches aimed at disrupting hypoxia‐driven signaling pathways or targeting specific metabolic pathways activated under low oxygen conditions hold promise for overcoming treatment resistance and improving patient survival [6, 36, 37].

With this background, we designed the present study to evaluate the relationship between hypoxia and metabolism in breast cancer model. Using systems approach along with experimental data, we evaluated the crosstalk between hypoxia and metabolic types in 2000 human breast tumors and unravel a context‐dependent relationship between hypoxia and metabolism.

## 2 Materials and methods

### 2.1 Hypoxia scoring of breast cancer patient cohorts and cell lines

The primary breast cancer cohort included in this study is composed of 1980 breast cancer patients with available survival and transcriptomic data from the METABRIC dataset. These data were obtained from European Genome‐phenome Archive (EGA) and details regarding the patients' consent, tissue collection, and processing have been previously published [38]. The METABRIC dataset was randomly split into discovery (*N* = 988) and validation (*N* = 992) cohorts, as described [39]. Subsequently, the hypoxia score of the tumors in each cohort were calculated. This was done by first extracting the expression levels of eight hypoxia‐responsive genes, namely, *DDIT4*, *LDHA*, *MXI1*, *NDRG1*, *P4HA1*, *PGK1*, *SLC2A1*, and *VEGFA*, that we have previously derived and validated in a panel of cancer cell lines, including breast cancer cell lines [11]. The median expression of each gene was calculated for each cohort independently, and subsequently, a value of 1 or −1 was given for each tumor in that cohort based on whether the expression of the gene is greater, or less than, the median, respectively. Thereafter, the values for the eight genes were added, giving rise to the hypoxia score. In addition to giving a hypoxia score, the cohorts were stratified into hypoxia score (HS) high (discovery *N* = 340; validation *N* = 360) and HS low (discovery *N* = 648; validation *N* = 632) based on whether their hypoxia score was greater than zero, or less than or equal to zero, respectively. The same strategy was used to give a hypoxia score to 1073 tumors from the BRCA‐TCGA publicly available dataset, for which expression data were obtained from UCSC Xena. Hypoxia scoring was additionally performed on 60 tumors from an independent dataset of breast cancer patients, (referred to as the metabolomics cohort), for which both expression and metabolomics data are available [40]. Finally, a panel of 56 breast cancer (BC) cell lines were given a hypoxia score using previously reported expression data [41]. Same breast tumor and cell line datasets were also scored using the 15‐gene Buffa signature [42]. The same scoring method described above was applied, this time using expression data from 15 genes (*ACOT7*, *ADM*, *ALDOA*, *CDKN3*, *ENO1*, *LDHA*, *MIF*, *MRPS17*, *NDRG1*, *P4HA1*, *PGAM1*, *SLC2A1*, *TPI1*, *TUBB6*, *VEGFA*).

### 2.2 Metabolic clustering of breast cancer patient cohorts and cell lines

This study integrated the previously reported metabolic types for the METABRIC (discovery and validation), TCGA‐BRCA, metabolomics, and BC cell lines datasets [39]. In brief, the metabolic types were derived by first using the *Pathifier* tool to calculate a deregulation score for 90 metabolic pathways in the discovery and validation cohort. The marked heterogeneity in the deregulation scores prompted sample clustering which stratified tumors into three distinct metabolic clusters, M1, M2, and M3. These clusters varied in their degree of metabolic deregulation, wherein the highest deregulation was exhibited by the M3 cluster and the lowest by M1 [39]. In this study, we used the identified clusters to capture the tumors' metabolic state for the discovery cohort (M1 *N* = 482; M2 *N* = 291; M3 *N* = 215); the validation cohort (M1 *N* = 611; M2 *N* = 122; M3 *N* = 259); the TCGA‐BRCA cohort (M1 *N* = 390; M2 *N* = 481; M3 *N* = 202); the metabolomics cohort (M1 *N* = 28; M2 *N* = 10; M3 *N* = 22); and the BC cell lines (M2 *N* = 20; M3 *N* = 36). Finally, the reported deregulation scores of the 90 metabolic pathways in the discovery and validation cohorts were used for correlation analysis with the hypoxia score. For the BC cell lines, we used the metabolic signatures of each cluster to predict cell line labels (M1/M2/M3) as described [39]. Surprisingly, only two types were identified in cell lines, M2 and M3, there were no cell lines representing the M1 metabolic cluster [39].

### 2.3 Hallmark pathways deregulation score

Cancer hallmark pathways (from MSigDB) consist of 50 curated gene sets marking key molecular processes relevant to cancer biology. The deregulation scores of these pathways in the discovery and validation cohorts were calculated using *Pathifier* algorithm, as described [39]. The higher the score of a pathway in a sample, the more deviated it is from the normal, and hence the more deregulated it is. Herein, the deregulation scores for 48 pathways were used for correlation analysis with the hypoxia score.

### 2.4 Tissue culture and treatment

The breast cancer cell lines MDA‐MB‐468 (RRID: CVCL_0419), MCF‐7 (RRID: CVCL_0031), BT474 (RRID: CVCL_0179), and T47D (RRID: CVCL_0553) and were purchased from the American Type Culture Collection (ATCC) and cultured in DMEM (Cat#: 11965092, Gibco, Thermo Fisher Scientific, Waltham, MA, USA) or RPMI (Cat#: 11875093, Gibco, Thermo Fisher Scientific) supplemented with 10% FBS (Cat#: A5256701, Gibco, Thermo Fisher Scientific) and 1% penicillin/streptomycin (Cat#: 15070063, Gibco, Thermo Fisher Scientific) as previously described [39, 43]. Cell lines had been authenticated by STR profiling (GenePrint^®^ 10 System, Cat#: B9510, Promega, Madison, WI, USA) and were tested and ensured to be mycoplasma free. For treatments, seeded cells were allowed 24 h to adhere followed by replacement of media with that containing 10 mm 2‐deoxyglucose (2DG) (Cat#: D8375, Sigma, St. Louis, MO, USA), or 10 μg·mL^−1^ C75 (Cat#: C5490, Sigma), or 100 μm leflunomide (Cat#: L5025, Sigma), or dimethyl sulfoxide (DMSO) (Cat#: D8418, Sigma) as control. Treatments were conducted for 24 h. Alternatively, for methionine deprivation 24 h post seeding media was replaced with methionine‐deficient media and cells were cultured for an additional 24 h. For all treatments, viability measurements were done using the CCK8 assay (Cat#: ab228554, Abcam, Cambridge, UK) according to manufacturer's instructions.

### 2.5 Survival and statistical analysis

To conduct survival analysis, RStudio v.1.3.1073 was used by first loading the survival, survminer, and dplyr packages. Kaplan–Meier plots were generated by comparing overall survival (OS), disease‐specific survival (DSS), and recurrence‐free survival (RFS) in the hypoxia score high and hypoxia low groups using log‐rank test. The Cox univariate and multivariate proportional‐hazards (PH) analysis were performed to evaluate prognostic significance of the hypoxia score and clinical variables. For all other statistical analysis, GraphPad Prism 10 was utilized. In the case of group comparisons, the Shapiro–Wilk test was first applied to assess the distribution of the data and in the case of a normal distribution, a parametric test was used, else a nonparametric one. For comparing two groups, the parametric two‐tailed, unpaired t‐test or the nonparametric Mann–Whitney test was applied. For more than two groups, the Kruskal–Wallis test was used with correction for multiple testing through the recommended Dunn's multiple comparison test. Regarding correlation analysis, Pearson correlation coefficients were computed. In all analyses, *P* < 0.05 was considered statistically significant. For data display, the online tool, Morpheus (Broad Institute) was additionally utilized to generate heatmaps of gene expression data or correlation coefficients.

## 3 Results

### 3.1 Hypoxia scoring of breast tumors reveals worse survival of patients with higher scores

**Fig. 1:**
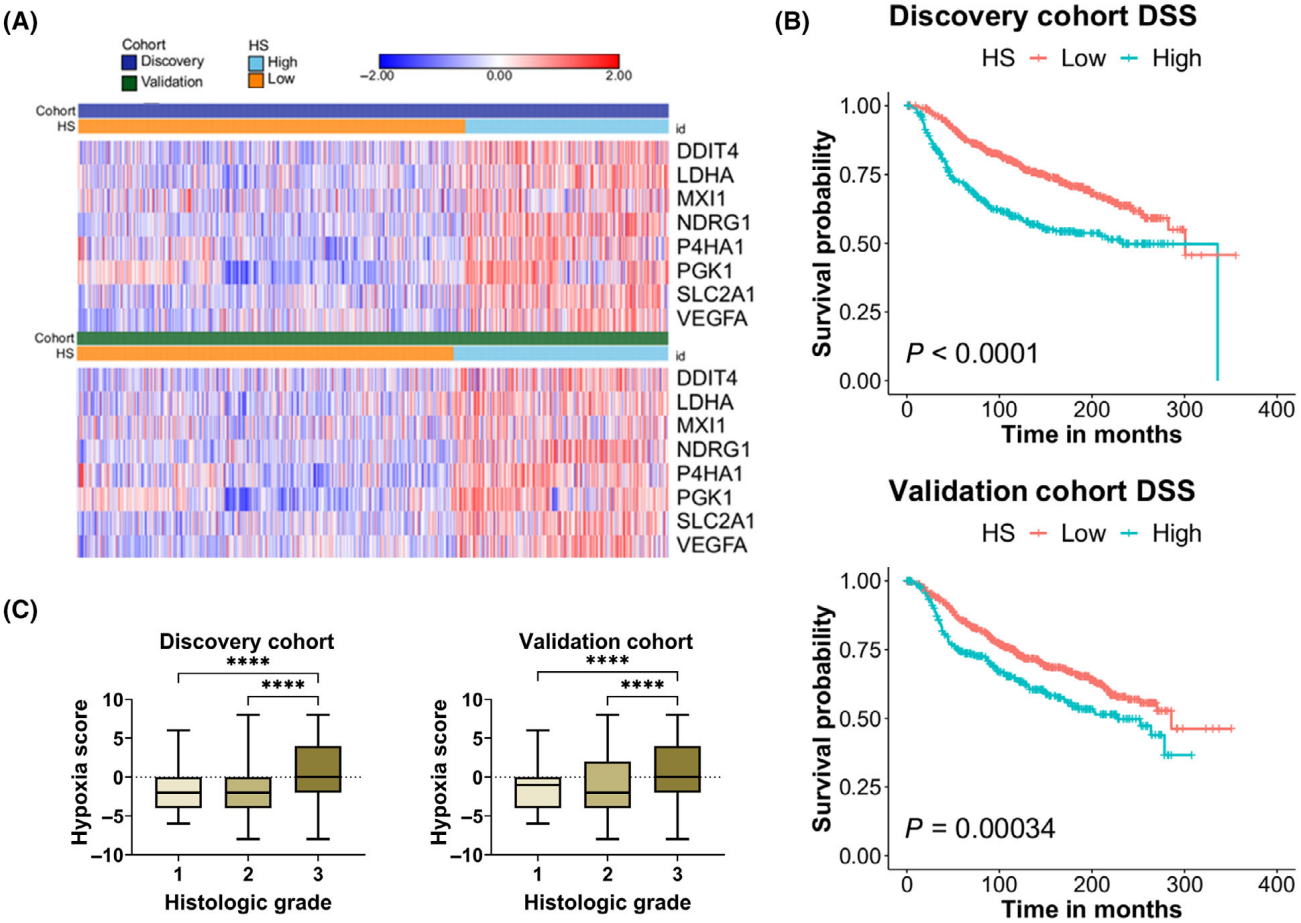
Hypoxia score is associated with worse survival in both discovery and validation cohorts of breast cancer patients. (A) Heatmaps of the expression levels of eight hypoxia‐related genes across patients stratified into high and low hypoxia score (HS). (B) Kaplan–Meier survival curves with log‐rank test comparing disease‐specific survival (DSS) in patients with high and low HS. (C) Box and whiskers plots of the median distribution of hypoxia scores across patients with different histologic grades. Whiskers represent minimum to maximum values per group. Comparison is based on Kruskal–Wallis test with Dunn's multiple comparisons correction. *P* < 0.05 considered statistically significant. ****P < 0.0001.

To evaluate how hypoxia relates to breast cancer patient outcome, we set out to score breast tumors of METABRIC dataset. To that end, we split the dataset into discovery and validation cohorts as described [39]. Thereafter, we employed an 8‐gene hypoxia signature to score tumors of both cohorts and labeled them as hypoxia score (HS) high and HS low to reflect their tumors' hypoxic state (Fig. 1A). We used the expression levels of 8 hypoxia‐related genes, namely *DDIT4*, *LDHA*, *MXI1*, *NDRG1*, *P4HA1*, *PGK1*, *SLC2A1*, and *VEGFA*, that we previously showed to be induced under hypoxic conditions in a panel of cancer cell lines, including those of breast cancer origin [11]. Importantly, patients with higher hypoxia score (HS high) significantly experienced worse disease‐specific, overall, and relapse‐free survival in both cohorts (Fig. 1B; Fig. S1a). The associations with survival were still significant when combined with clinical covariates of disease stage, grade, and PAM50 classification in a multivariate analysis (Fig. S1b; Table S1). Therefore, hypoxia, as determined by our signature, is an independent prognostic marker of survival in the queried breast cancer datasets. Furthermore, tumors histologically classified as grade 3 and thereby having more advanced disease, displayed significantly higher hypoxia scores compared to those of grades 1 and 2, respectively (Fig. 1C). These results indicate that the hypoxic state of tumors is predictive of patient outcomes and disease aggressiveness.

### 3.2 Hypoxia is tightly linked with metabolic deregulation

**Fig. 2:**
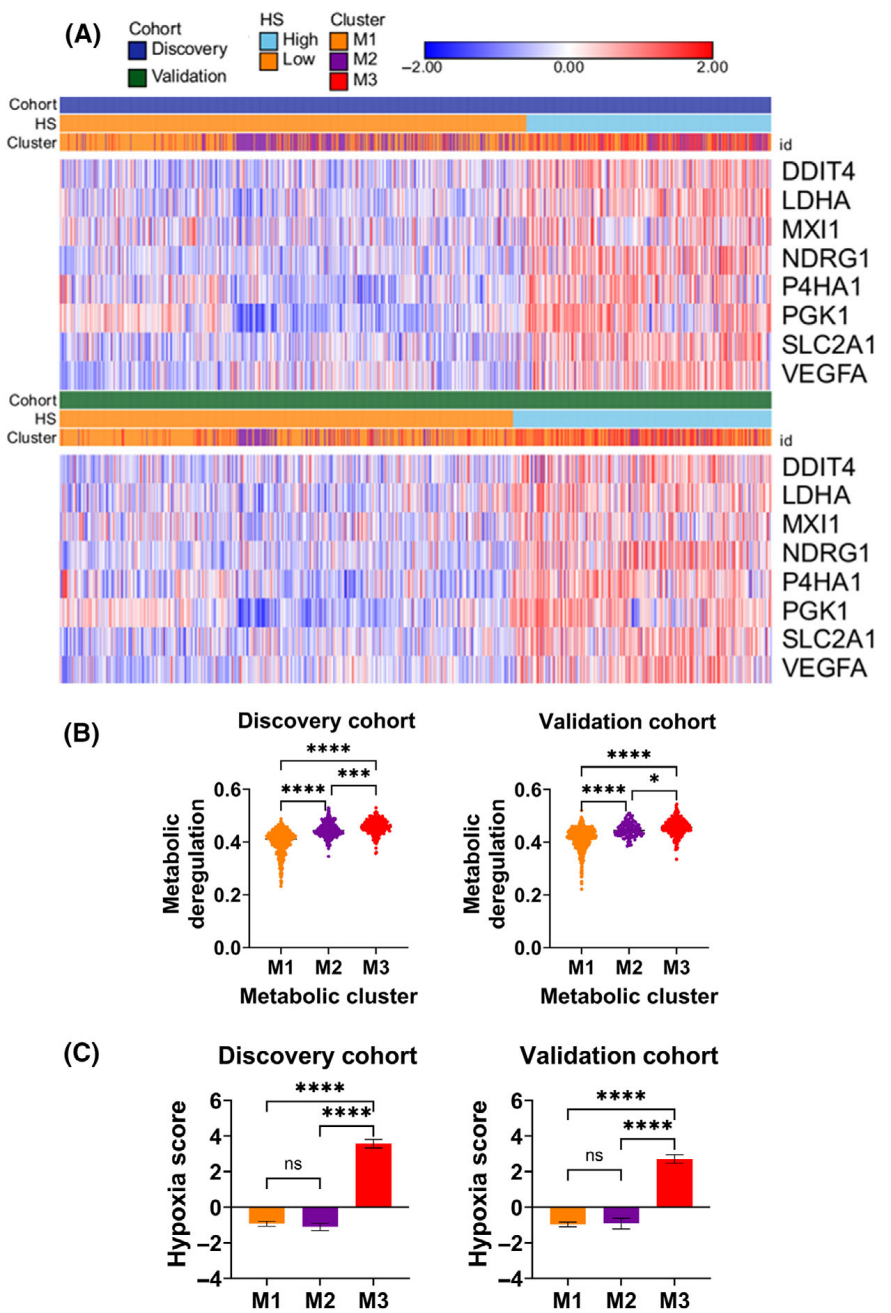
Hypoxia score is highest in metabolic cluster M3 in both discovery and validation cohorts. (A) Heatmaps of the expression levels of eight hypoxia‐related genes across patients stratified into high and low hypoxia score (HS) as well as metabolic clusters M1, M2, and M3. (B) Violin plots of the metabolic deregulation scores in M1 compared to M2 and M3 metabolic clusters. (C) Bar graphs of the mean and standard error mean of the hypoxia scores in M1 compared to M2 and M3 metabolic clusters. Comparisons based on Kruskal–Wallis nonparametric test with Dunn's multiple comparisons correction. *P* < 0.05 considered statistically significant. ****P < 0.0001; ***P = 0.0002; *P = 0.0343; ns, not significant.

To understand how hypoxia relates with metabolism in breast tumors, we assessed the distribution of hypoxia score high and low tumor samples across metabolic types which we previously identified [39]. Briefly, we stratified breast tumors based on their global metabolic profile using a pathway‐based systems approach and identified three metabolic types, namely M1, M2, and M3 (Fig. 2A). We used *Pathifier* to calculate deregulation scores of 90 metabolic pathways in METABRIC breast tumors by feeding the gene expression values into the algorithm [39]. Notably, M3 type exhibited highest metabolic deregulation (Fig. 2B). Next, using hypoxia score metric, we calculated the average hypoxia score for each of the three types. While M1 and M2 showed similar trends with regards to hypoxia score, M3 tumors exhibited a remarkable difference with highest hypoxia score, both in discovery and validation cohorts (Fig. 2C). This behavior coincided with the highest metabolic deregulation in M3‐type tumors (Fig. 2B), suggesting a strong association between hypoxia and metabolism. Of note, we were able to reproduce this relationship trend between HS and metabolic types in two other independent cohorts (Fig. S2a). Interestingly, breast cancer cell lines also showed this pattern whereby M3 cluster had significantly higher HS (Fig. S2b). Of note, these findings were reproduced with an independent 15‐gene Buffa hypoxia signature [42] (Fig. S2c). To sum up, these results demonstrate that hypoxia and metabolism in breast tumors are tightly linked hallmarks.

### 3.3 Correlation of hypoxia and metabolic deregulation is context specific

**Fig. 3:**
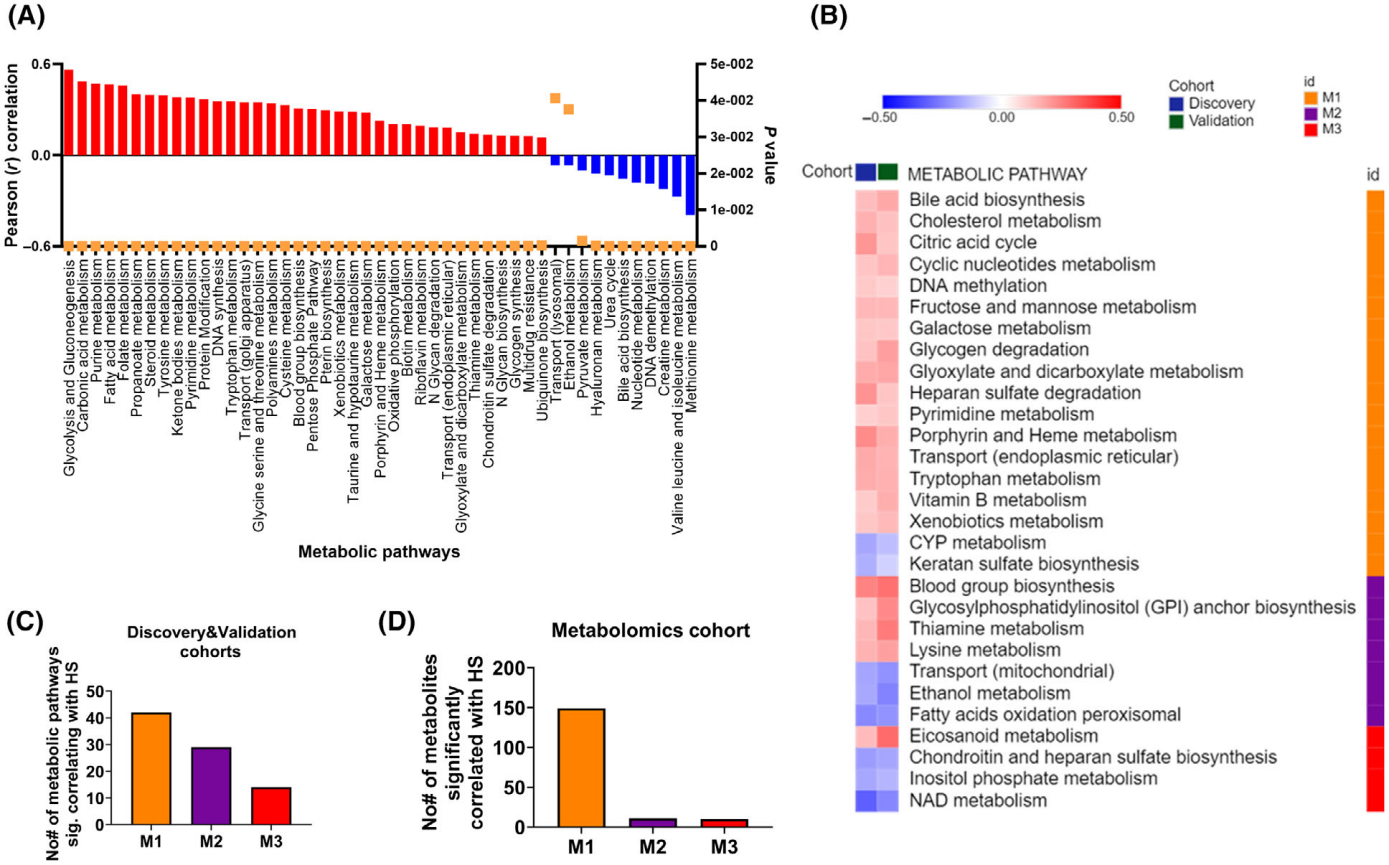
Hypoxia score is correlated with deregulation of metabolic pathways. (A) Waterfall plot of the Pearson (*r*) correlation coefficients between hypoxia score and metabolic deregulation score of metabolic pathways along with the corresponding *P*‐values in the discovery cohort. (B) Heatmap of Pearson (*r*) correlation coefficients reflecting significant positive (red) or negative (blue) correlation between hypoxia score and deregulation scores of metabolic pathways in the discovery and validation cohorts. (C) Number of metabolic pathways significantly correlated with hypoxia score (HS) across metabolic clusters in both the discovery and validation cohorts. (D) Number of metabolites significantly correlated with HS across metabolic clusters in the metabolomics cohort.

It is well established that tumor cells in hypoxia undergo various adaptive changes often involving alterations in cellular metabolism to support the energy demands and maintain their growth under low oxygen conditions. Herein, we evaluated the correlation between the hypoxia score and 90 metabolic pathways representing global metabolism. We found a strong positive correlation between HS and 36 metabolic pathways and negative correlation of 11 pathways, consistently in the discovery and validation cohorts (Fig. 3A; Fig. S3a). Among the most deregulated pathways in tumors with high hypoxia score were pathways of glycolysis and gluconeogenesis, as well as carbonic acid metabolism, fatty acid metabolism, purine, and pyrimidine metabolism; on the other hand, methionine metabolism was among the most deregulated in tumors with low hypoxia score. Interestingly, the deregulation score of the metabolic pathways positively correlated with HS were highest in the M3 metabolic cluster. On the other hand, for methionine metabolism that is negatively correlated with HS, it was lowest in M3 in both discovery and validation cohorts (Fig. S3b). To further refine the analysis, we evaluated the correlation between hypoxia and global metabolism in the three metabolic types across discovery and validation cohorts. The reported pathways that were found to strongly correlate with hypoxia score were also found to be commonly correlated across the three clusters (Fig. S4b; Table S2). Nonetheless, the overall correlation pattern between HS and metabolic pathways was unique to each cluster (Fig. 3B). Notably, HS correlated with the highest number of metabolic pathways in the M1 cluster. This number kept decreasing with the least number of metabolic pathways showing significant correlation with HS in M3 (Fig. 3C; Fig. S4a). This surprising pattern prompted us to reproduce the correlation analysis in an independent dataset of 60 tumors with metabolite data, and a similar trend was observed (Fig. 3D; Table S3). That is, the highest number of metabolites from M1 cluster and least in M3 showed significant correlation with HS. The consistent correlation pattern at transcriptional and metabolite levels in tumors of independent cohorts not only reflects robustness of observations but also highlights a context‐dependent relation between hypoxia and metabolism. Furthermore, these findings suggest that metabolic adaptation is influenced early on by the presence of hypoxia in the tumor microenvironment.

### 3.4 Experimental inhibition of metabolic pathways validated the correlation between hypoxia and metabolic pathways

**Fig. 4:**
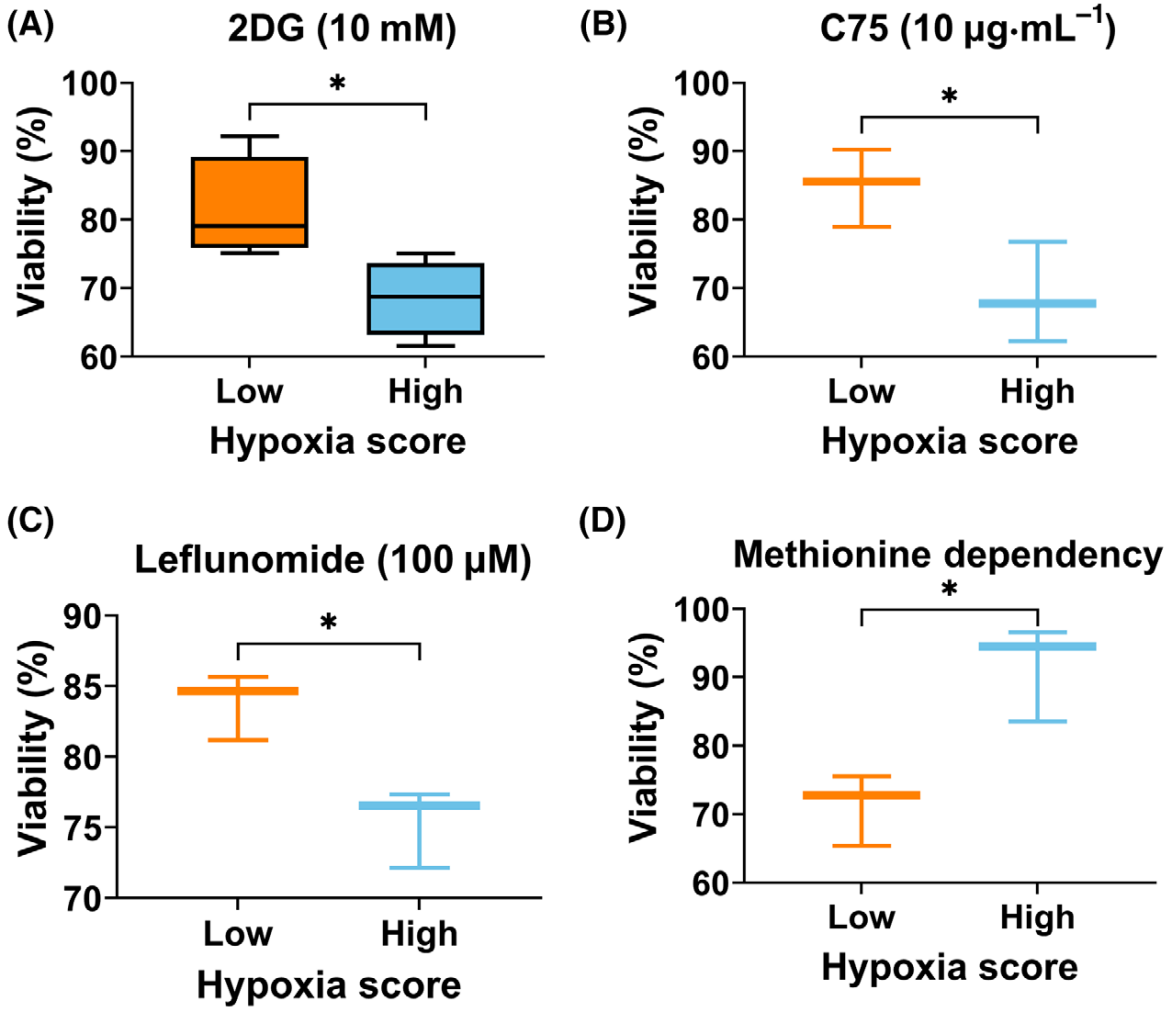
Sensitivity of breast cancer cell lines to the availability of metabolites is associated with hypoxia score. (A–D) Box and whiskers plot of the median viability of low hypoxia score cells (MCF‐7) compared to high hypoxia score cells (MDA‐MB‐468), upon treatment with the glycolysis inhibitor 2‐deoxyglucose (2DG) (A), fatty acid metabolism inhibitor (C75) (B), pyrimidine synthesis inhibitor (Leflunomide) (C), or deprivation of methionine (D) for 24 h. Whiskers represent minimum to maximum values per group obtained from three replicates. Comparisons based on unpaired *t*‐test and *P*‐value < 0.05 considered statistically significant. *P > 0.0021.

To experimentally validate the above findings, breast cancer cell lines were first classified using the eight‐gene hypoxia signature as described in the Materials and Methods section. We then treated low hypoxia score cell line MCF‐7 and high hypoxia score cell line MDA‐MB‐468 with inhibitors of metabolic pathways found to be positively correlated with hypoxia score. For example, glycolysis inhibitor 2‐deoxyglucose (2DG), fatty acid synthesis inhibitor (C75), and pyrimidine synthesis inhibitor (leflunomide) were used at indicated concentrations for 24 h. Upon determining cell viability, the three agents were significantly more effective in the high hypoxia score cell line, MDA‐MB‐468, compared to the low hypoxia score cell line, MCF‐7 (Fig. 4A–C). Similar results were recapitulated in additional cell lines (Fig. S5). These findings are consistent with gene expression‐based analysis presented in Fig. 3A showing that hypoxia score is positively correlated with glycolysis, as well as pyrimidine synthesis and fatty acid metabolism. We next tested methionine metabolism dependency of the cells, as this pathway was negatively correlated with hypoxia score (Fig. 3A). The reduced viability of MCF‐7 cells compared to MDA‐MB‐468 upon methionine deprivation revealed a higher dependency of low hypoxia scoring cells on methionine metabolism (Fig. 4D). This again is consistent with the negative correlation of hypoxia score with methionine metabolism. In summary, these results validate gene expression‐based correlation analysis and additionally highlight the relevance of metabolic‐gene expression in reflecting experimental metabolism.

### 3.5 Hypoxia score correlates with biological pathways in a context‐specific manner

**Fig. 5:**
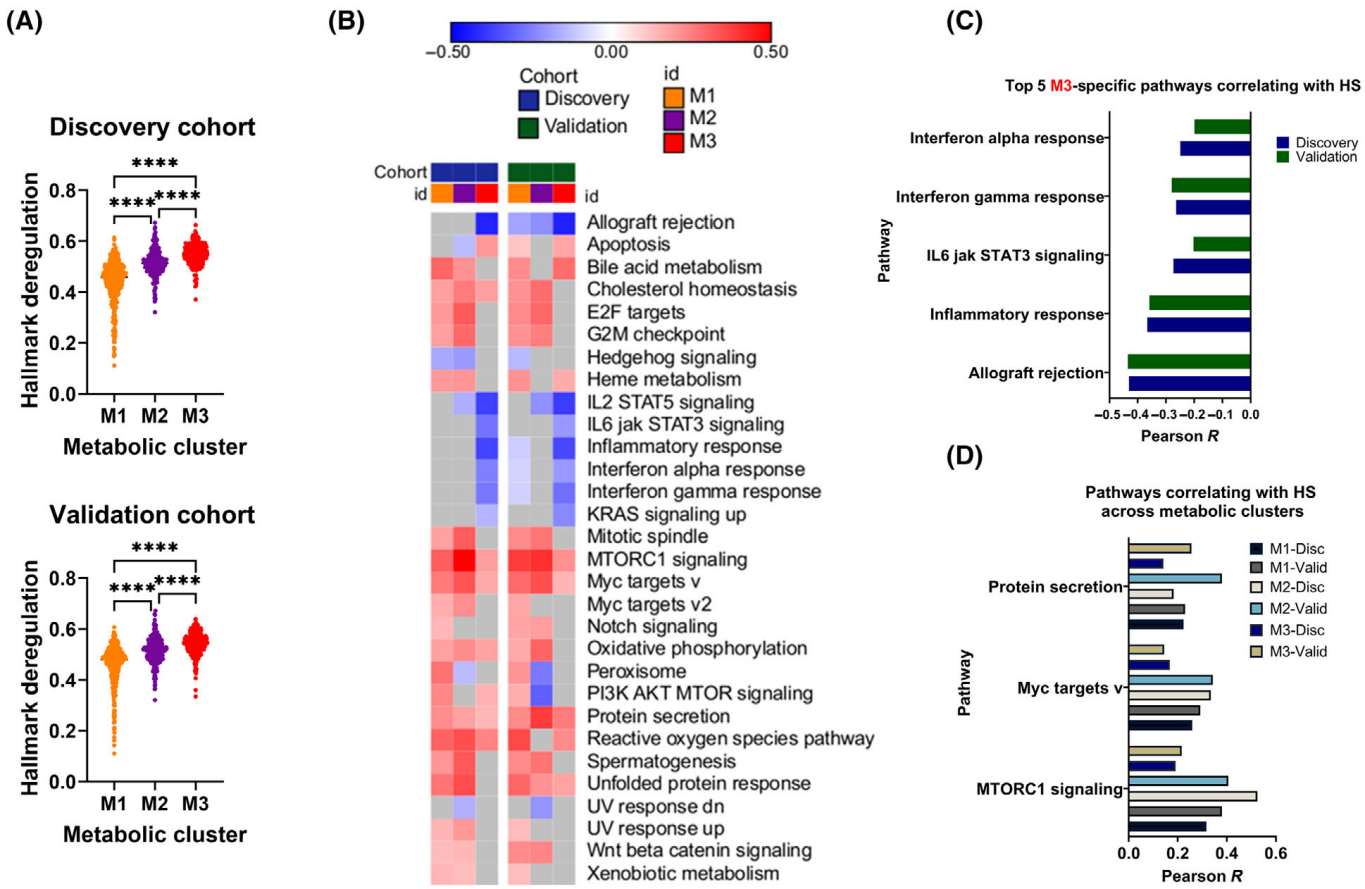
Landscape of correlations between hypoxia score and hallmark signaling pathways across metabolic clusters. (A) Violin plots of the hallmark deregulation scores in M1 and M2 compared to M3 in the discovery and validation cohorts. (B) Heatmap of Pearson (*r*) correlation coefficients reflecting significant positive correlation (red), negative correlation (blue), or no significant correlation (gray) between hypoxia score and deregulation scores of hallmark signaling pathways across metabolic clusters in the discovery and validation cohorts. (C, D) Pearson (*r*) correlation coefficients of pathways significantly correlated with hypoxia score (HS) that are top 5 in M3 (C), or common to M1, M2, and M3 (D). Comparisons based on Kruskal–Wallis nonparametric test with Dunn's multiple comparisons correction. *P*‐value < 0.05 considered statistically significant. ****P < 0.0001.

In order to understand the association of hypoxia with cancer hallmark pathways, deregulation scores for 48 MSigDb pathways [44] in M1, M2, and M3 samples of discovery and validation cohorts were calculated as described in Materials and Methods. The average deregulation score of the hallmark pathways was found to be highest in the M3 cluster in both cohorts (Fig. 5A). Thereafter, a correlation analysis was performed between hypoxia and deregulation scores in each of the three metabolic types. The relation of hypoxia and cancer pathways showed a context‐specific pattern with an overall unique correlation fingerprint in each metabolic type (Fig. 5B; Fig. S6). Positive correlations between Notch signaling and PI3K‐AKT–mTOR signaling with the hypoxia score were unique to the M1 metabolic cluster (Fig. S6). On the other hand, immune response‐related pathways (allograft rejection, cytokine signaling, and inflammatory response) showed a negative correlation with the hypoxia score in M3 cluster only (Fig. 5C; Fig. S6). In addition, pathways of protein secretion, Myc targets and mTORC1 signaling were found to be consistently correlated with hypoxia score across all metabolic types (Fig. 5D). These results highlight how biology of tumor metabolic types and its interplay with hypoxia is heterogenous.

## 4 Discussion

Hypoxia plays a significant role in the metabolism regulation of solid tumors, influencing various aspects of cancer progression and treatment resistance. However, it should be noted that metabolism is highly complex and understanding the impact of hypoxia on global metabolism is critical to gain deeper insights into the interplay between hypoxia and tumor metabolism. With the advent of high‐throughput technologies and generation of big data, systems‐level understanding of biological processes has provided useful insights pertinent to basic and clinical research in cancer. Accordingly, we attempted to explore how hypoxia relates to the global metabolic landscape to gain a better understanding of crosstalk between these two key processes in the context of breast cancer.

As an important tumor microenvironmental factor, hypoxia is known to affect a variety of clinical indicators including patient outcome, confirmed by our 8‐gene hypoxia signature‐based scoring (Fig. 1). Hypoxia predicting a worse patient prognosis in both discovery and validation cohorts underlines how poor oxygen availability benefits cancer possibly by selecting aggressive clones that adapt metabolically to hypoxic condition (Figs 1, 2). Tumor microenvironmental features, like hypoxia, nutrient availability, are known to select aggressive clones during tumor evolution [45, 46]. In fact, hypoxia is a critical selection driver and shapes metabolic programs engaged by evolving tumor cells [47].

Studying the relation between hypoxia and metabolism in three metabolic types of breast tumors provided a setting for a more nuanced understanding of hypoxia‐metabolism nexus. Evidently, not all types of breast cancer showed a consistent relationship with hypoxia; in this respect, only M3 type demonstrated an outstanding association with hypoxia (Fig. 2). Since M3 is also the most metabolically deregulated type, it could be inferred that hypoxia strongly relates to metabolic deregulation. Additionally, M3 is primarily populated by high‐grade tumors [39], therefore, it is likely that hypoxia also predicts clinical aggressiveness of breast tumors. The most profound effect of hypoxia on cancer cells is their reliance on glycolysis leading to increased glucose consumption and lactate production [20]. This metabolic shift not only provides energy but also generates metabolic intermediates necessary for biosynthesis, supporting tumor growth, and proliferation. However, we provided evidence that the impact of hypoxia on metabolism is not restricted to glycolysis and goes beyond (Fig. 3). Moreover, the relationship between hypoxia and metabolism was highly contextual as suggested by an overall unique fingerprint of correlation between hypoxic score and 90 pathways correlation pattern in three metabolic types (Fig. 3). The experimental data showing inhibition of different metabolic pathways in cell lines with different hypoxia score further confirmed the contextual relationship between hypoxia and metabolism (Fig. 4). For instance, pyrimidine metabolism inhibition by leflunomide showed that high hypoxia score cell lines are more sensitive to its inhibition compared to ones with lower hypoxia score (Fig. 4). On the other hand, methionine metabolism showed opposite dependencies, consistent with correlation analysis (Fig. 4). These results representatively point to differential metabolic pathways that are engaged depending on the hypoxic state. Further, experimental metabolic inhibition also demonstrates how hypoxia‐metabolism connection can shape response to drugs (Fig. 4).

It is widely admitted that the altered metabolism contributes to therapeutic resistance in solid tumors [23] and hypoxic regions within tumors often exhibit reduced sensitivity to chemotherapy and radiation therapy [6]. The metabolic type‐dependent relationship of hypoxia with metabolism along with validation on cancer cell lines suggests that targeting specific metabolic pathways in relation to hypoxia may overcome treatment resistance and improve patient outcomes. The maximal number of metabolic pathways and metabolites in M1 showing significant correlation with hypoxia, followed by M2 and least in M3, suggests that M1 tumors are most sensitive to the impact of hypoxic stress (Fig. 3). In other words, metabolic adaptation is shaped early on by hypoxia and that metabolism and hypoxia crosstalk contributes to tumor aggressiveness and poor patient outcome in breast cancer (Figs 1, 2, 3). Furthermore, the negative correlation of high hypoxia score M3 with immune response pathways strongly suggests that compromised antitumor immunity in these tumors and high hypoxia and metabolic deregulation might be associated to immune escape (Fig. 5). Low immune activity in high hypoxia score tumors may explain the poor prognosis (Fig. 1). From a different perspective, excessive metabolic deregulation in high hypoxia score M3 tumors may explain lower antitumor immunity as metabolic activity has been implicated in immune evasion in M3‐like breast cancers [18].

## 5 Conclusions

In conclusion, the results of the current study reveal how hypoxia relates with global metabolism and that this relation is context specific. In addition, results also suggest that drugs that target specific metabolic pathways, such as inhibitors of pyrimidine metabolism may disrupt the metabolic adaptations of hypoxic cancer cells. Therefore, combining metabolic inhibitors with conventional cancer treatments or other targeted therapies may synergistically enhance treatment efficacy and overcome therapeutic resistance in hypoxic tumors. Utilizing patient‐specific metabolic profiling and biomarkers to identify metabolic vulnerabilities in hypoxic tumors may presumably guide the selection of optimal treatment strategies. Tailoring therapies based on the unique metabolic characteristics of individual tumors may improve treatment outcomes and reduce the likelihood of resistance development.

## Conflict of interest

The authors declare no conflict of interest.

## Author contributions

MAI and SC were involved in conception and design. RAK and MAI conducted data analysis, curated the results, and have written the original draft of the manuscript. All authors critically read, reviewed, and agreed to the submission of the manuscript.

## Peer review

The peer review history for this article is available at https://www.webofscience.com/api/gateway/wos/peer‐review/10.1002/1878‐0261.13762.

## Supporting information


**Fig. S1.** Association of hypoxia score with survival.
**Fig. S2.** Distribution of hypoxia scores among metabolic clusters.
**Fig. S3.** Metabolic pathways significantly correlated with the hypoxia score and the metabolic deregulation scores of select pathways across the metabolic clusters.
**Fig. S4.** Correlation between hypoxia score and metabolic deregulation.
**Fig. S5.** Viability of breast cancer cell lines to the availability of metabolites based on hypoxia score.
**Fig. S6.** Correlation of deregulation scores of cluster‐specific hallmark signaling pathways with hypoxia scores.

**Table S1.** Univariate and Multivariate COX PH analysis in METABRIC discovery and validation cohorts.
**Table S2.** Metabolic pathways correlated with hypoxia score across the three metabolic clusters.
**Table S3.** Metabolites significantly correlated with hypoxia score across the metabolic clusters.

## Data Availability

No material was generated in this study. The METABRIC data were retrieved from the European Genome‐Phenome Archive (EGAD00010000210, EGAD00010000211). The BRCA‐TCGA data were obtained from UCSC Xena and the metabolomics data from gene expression omnibus (GSE37751).
