# Supplementary material for: Integrative systems‐level analysis reveals a contextual crosstalk between hypoxia and global metabolism in human breast tumors
Source: Mol Oncol. 2024 Dec 27;19(6):1725–36. doi: 10.1002/1878-0261.13762 (PMC12161489; doi:10.1002/1878-0261.13762)
Supplement: Supplementary file 1 — Fig. S1. Association of hypoxia score with survival. Fig. S2. Distribution of hypoxia scores among metabolic clusters. Fig. S3. Metabolic pathways significantly correlated with the hypoxia score and the metabolic deregulation scores of select pathways across the metabolic clusters. Fig. S4. Correlation between hypoxia score and metabolic deregulation. Fig. S5. Viability of breast cancer cell lines to the availability of metabolites based on hypoxia score. Fig. S6. Correlation of deregulation scores of cluster‐specific hallmark signaling pathways with hypoxia scores. [file MOL2-19-1725-s002.pdf]

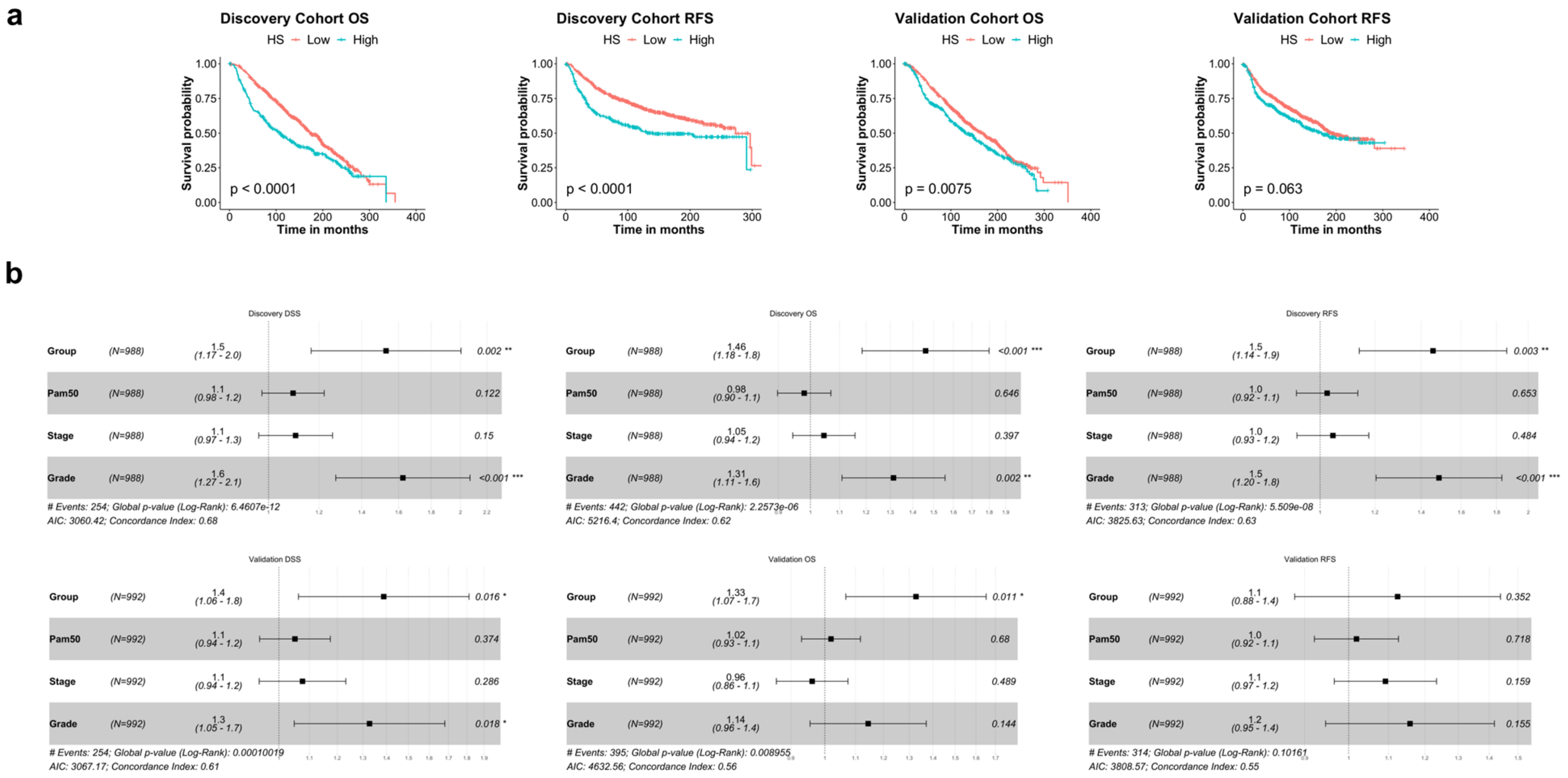

Supplementary Fig. 1: Association of hypoxia score with survival. **a** Kaplan-Meier survival curves with log-rank test comparing overall survival (OS) and recurrence-free survival (RFS) in patients with low and high hypoxia score (HS) in the discovery and validation cohorts. **b** Multivariate COX PH Forest plots showing hazard ratio of DSS, OS and RFS in the discovery and validation cohorts, considering the 8-gene HS and other clinical covariates. P-value < 0.05 considered statistically significant.

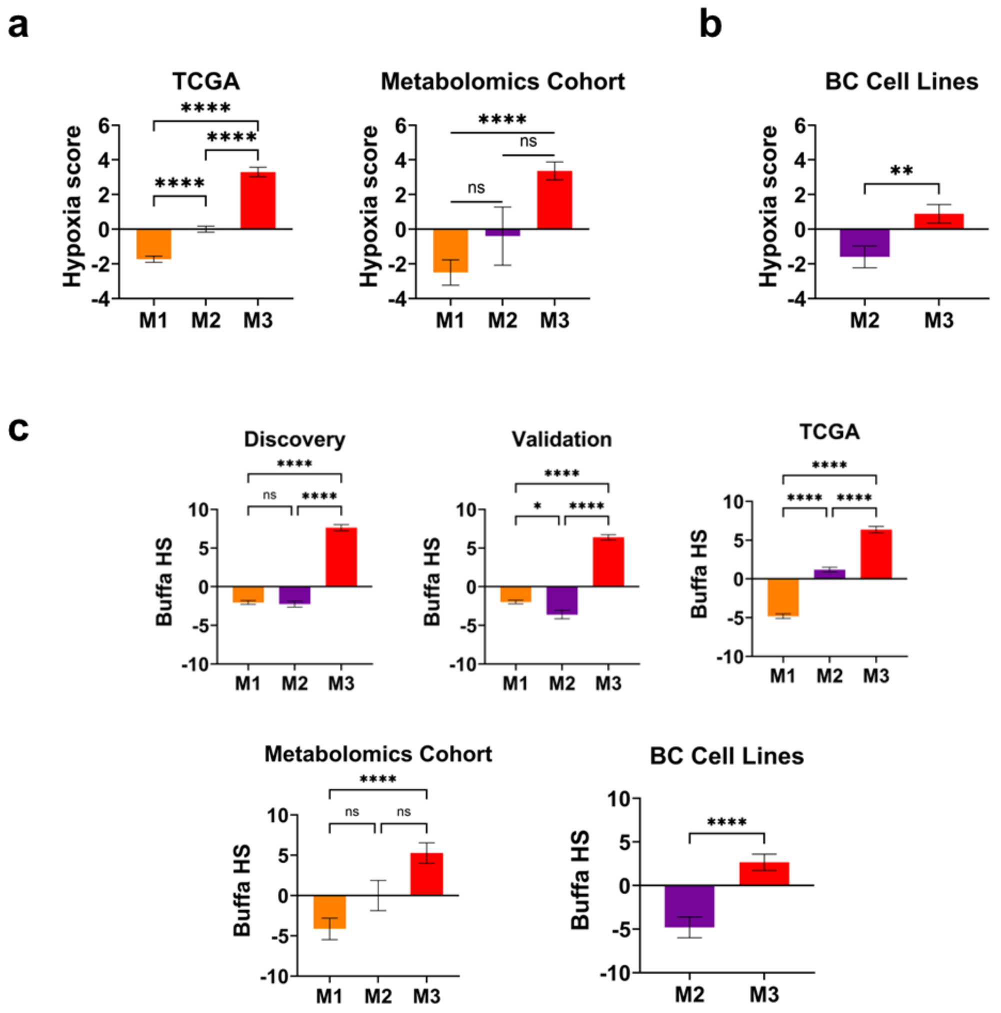

Supplementary Fig. 2: Distribution of hypoxia scores among metabolic clusters. Bar graphs of the mean and standard error mean of the hypoxia scores compared across metabolic clusters using the 8-gene hypoxia signature (**a,b**) or the 15-gene Buffa signature (**c**). There is no representation of M1 breast cancer (BC) cell lines, therefore this cluster is not included. Comparisons of three groups is based on Kruskal-Wallis non-parametric test with Dunn's multiple comparisons correction. Comparisons of two groups is based on unpaired t-test (**b**) or Mann Whitney test (**c**). P-value < 0.05 considered statistically significant. ns: not significant; *: p<0.032; **: p< 0.0021; ****: p<0.0001.

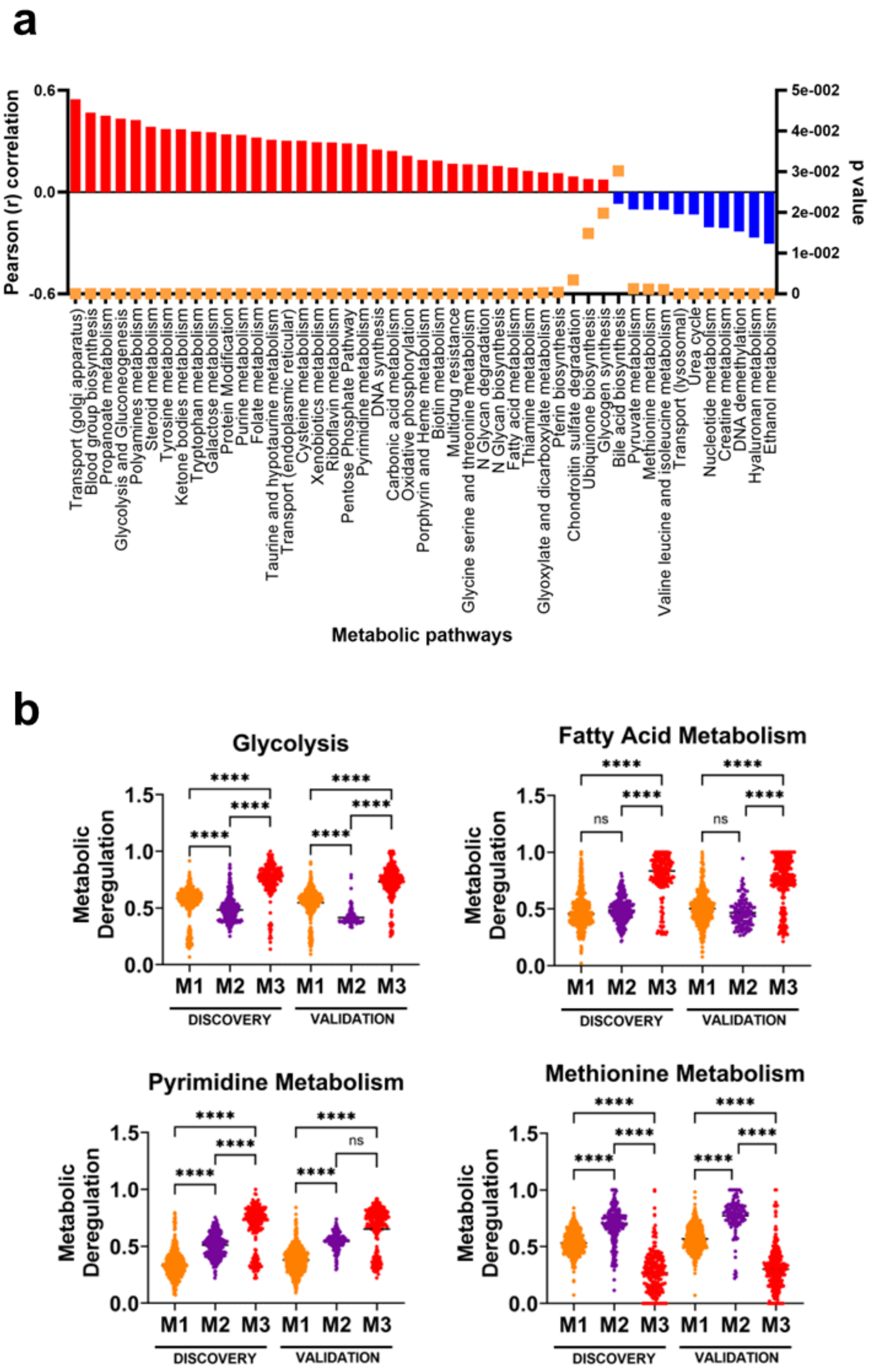

Supplementary Fig. 3: Metabolic pathways significantly correlated with the hypoxia score and the metabolic deregulation scores of select pathways across the metabolic clusters. **a** Waterfall plot of the Pearson (r) correlation coefficients between hypoxia score and metabolic deregulation score of metabolic pathways along with the corresponding p-values in the validation cohort. **b** deregulation scores of pathways strongly correlated with hypoxia score across metabolic clusters in the discovery and validation cohorts. Comparisons based on Kruskal-Wallis non-parametric test with Dunn's multiple comparisons correction. P-value < 0.05 considered statistically significant. ns: not significant; ****: p<0.0001.

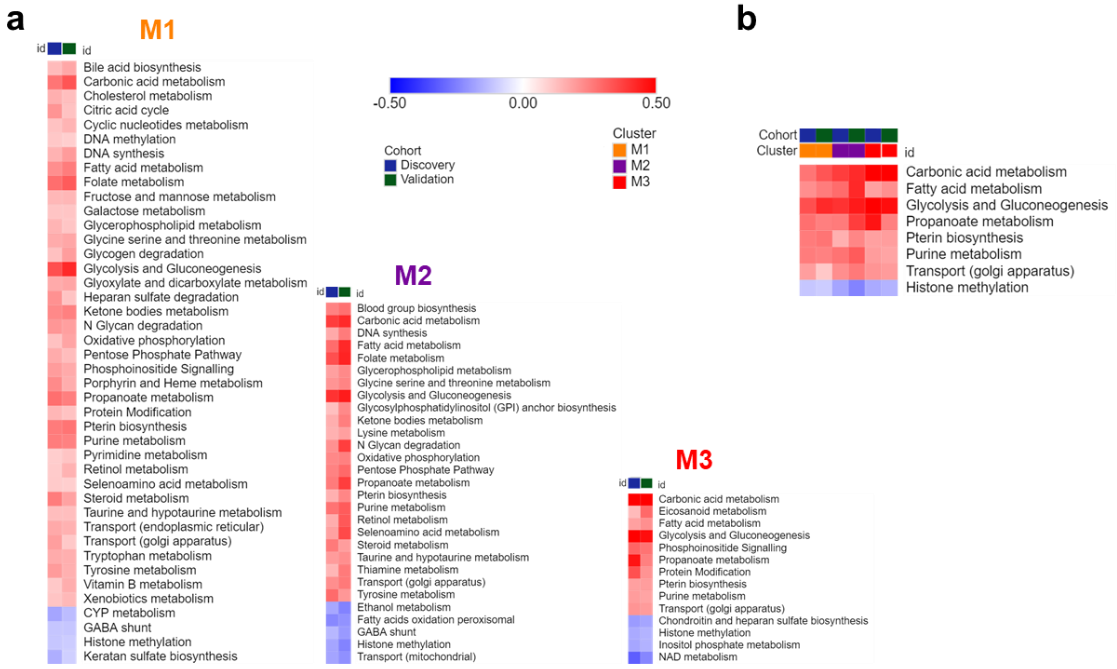

Supplementary Fig. 4: Correlation between hypoxia score and metabolic deregulation. **a** Heatmaps of significant Pearson correlation coefficients between metabolic deregulation scores of metabolic pathways and hypoxia scores in both discovery and validation cohorts across M1, M2 and M3 metabolic clusters. **b** Heatmaps of significant Pearson correlation coefficients between metabolic deregulation scores of metabolic pathways and hypoxia scores common to all three metabolic clusters.

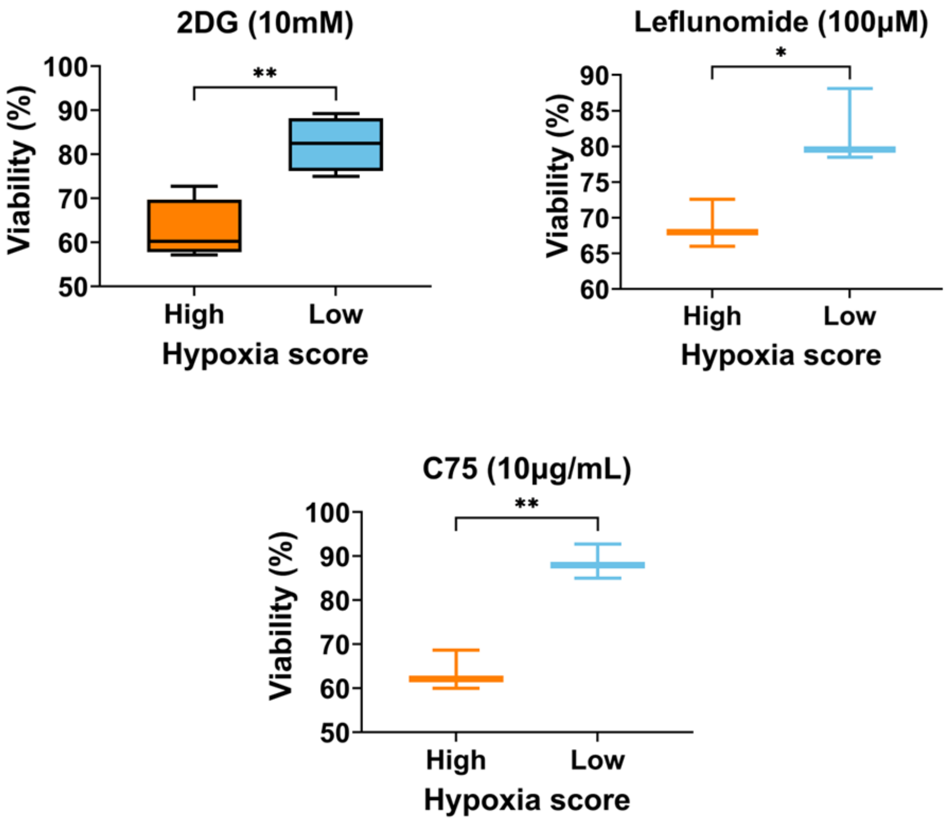

Supplementary Fig. 5: Viability of breast cancer cell lines to the availability of metabolites based on hypoxia score. Box and whiskers plot of the median viability of low hypoxia score cells (T47D) compared to high hypoxia score cells (BT474), upon treatment with the glycolysis inhibitor 2-deoxyglucose (2DG), pyrimidine synthesis inhibitor (Leflunomide) and fatty acid metabolism inhibitor (C75) for 24 hours. Whiskers represent minimum to maximum values per group obtained from three replicates. Comparisons based on unpaired t-test and p-value<0.05 considered statistically significant. *: p>0.0021; **: p<0.0021.

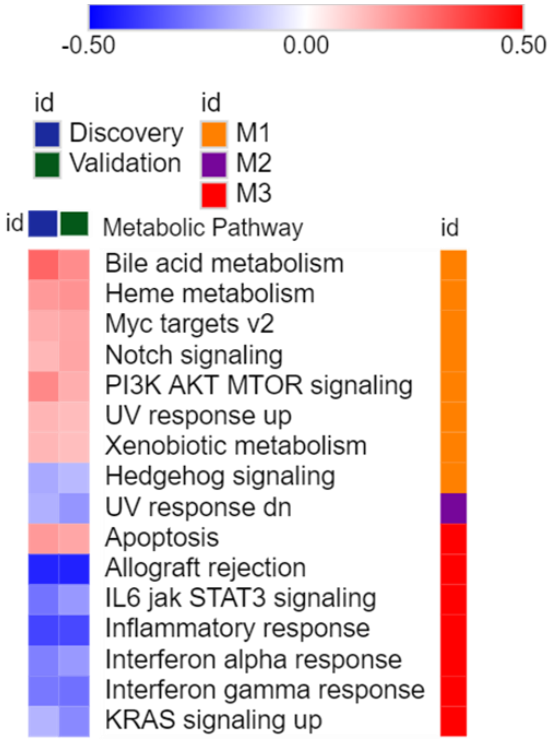

Supplementary Fig. 6: Correlation of deregulation scores of cluster-specific hallmark signaling pathways with hypoxia scores. Heatmap of Pearson (r) correlation coefficients reflecting significant positive correlation (red) and negative correlation (blue) between hypoxia score and deregulation scores of hallmark signaling pathways specific to each metabolic cluster in the discovery and validation cohorts.
