# Supplementary material for: Integrative systems‐level analysis reveals a contextual crosstalk between hypoxia and global metabolism in human breast tumors
Source: Mol Oncol. 2024 Dec 27;19(6):1725–36. doi: 10.1002/1878-0261.13762 (PMC12161489; doi:10.1002/1878-0261.13762)
Supplement: Supplementary file 2 — Table S1. Univariate and Multivariate COX PH analysis in METABRIC discovery and validation cohorts. Table S2. Metabolic pathways correlated with hypoxia score across the three metabolic clusters. Table S3. Metabolites significantly correlated with hypoxia score across the metabolic clusters. [file MOL2-19-1725-s001.pdf]

Supplementary Table 1. Univariate and Multivariate COX PH analysis in METABRIC discovery and validation cohorts.

	Disease Specific Survival				Overall Survival				Recurrence Free Survival			
	Univariate HR [95% CI]	P-value	Multivariate HR [95% CI]	P-value	Univariate HR [95% CI]	P-value	Multivariate HR [95% CI]	P-value	Univariate HR [95% CI]	P-value	Multivariate HR [95% CI]	P-value
**Discovery (n=988)**												
Hypoxia group (high score vs low score)	2 [1.6-2.5]	**<0.001**	1.5 [1.17-2]	**0.002**	1.5 [1.3-1.8]	**<0.001**	1.46 [1.18-1.8]	**<0.001**	1.7 [1.4-2.1]	**<0.001**	1.5 [1.14-1.9]	**0.003**
PAM50 (Basal vs Her2 vs LumB vs LumA vs Normal-like)	1.3 [1.2-1.4]	**<0.001**	1.1 [0.98-1.2]	0.122	1.1 [1-1.2]	**0.004**	0.98 [0.9-1.1]	0.646	1.2 [1.1-1.3]	**<0.001**	1 [0.92-1.1]	0.653
Stage (III&IV vs II vs I vs 0)	1.2 [1-1.3]	**0.015**	1.1 [0.97-1.3]	0.15	1.1 [0.99-1.2]	0.077	1.05 [0.94-1.2]	0.397	1.1 [0.98-1.2]	0.118	1 [0.93-1.2]	0.484
Grade (1 vs 2 vs 3)	1.9 [1.6-2.4]	**<0.001**	1.6 [1.27-2.1]	**<0.001**	1.4 [1.2-1.6]	**<0.001**	1.31 [1.11-1.6]	**0.002**	1.7 [1.4-2]	**<0.001**	1.5 [1.2-1.8]	**<0.001**
**Validation (n=992)**												
Hypoxia group (high score vs low score)	1.5 [1.2-1.8]	**<0.001**	1.4 [1.06-1.8]	**0.016**	1.3 [1.1-1.5]	**0.008**	1.33 [1.07-1.7]	**0.011**	1.2 [0.99-1.5]	0.064	1.1 [0.88-1.4]	0.352
PAM50 (Basal vs Her2 vs LumB vs LumA vs Normal-like)	1.2 [1.1-1.3]	**<0.001**	1.1 [0.94-1.2]	0.374	1.1 [1-1.1]	**0.043**	1.02 [0.93-1.1]	0.68	1.1 [1-1.2]	0.054	1 [0.92-1.1]	0.718
Stage (III&IV vs II vs I vs 0)	1.1 [0.97-1.3]	0.119	1.1 [0.94-1.2]	0.286	0.99 [0.88-1.1]	0.806	0.96 [0.86-1.1]	0.489	1.1 [0.98-1.2]	0.112	1.1 [0.97-1.2]	0.159
Grade (1 vs 2 vs 3)	1.5 [1.3-1.8]	**<0.001**	1.3 [1.05-1.7]	**0.018**	1.2 [1-1.4]	**0.019**	1.14 [0.96-1.4]	0.144	1.2 [1-1.4]	**0.012**	1.2 [0.95-1.4]	0.155

HR: hazard ratio; CI: confidence interval; Bold are significant p-values (p-value<0.05)

Supplementary Table 2. Metabolic pathways correlated with hypoxia score across the three metabolic clusters.

SN	Metabolic Pathway	M1				M2				M3			
		Discovery		Validation		Discovery		Validation		Discovery		Validation	
		Pearson R	P-value	Pearson R	P-value	Pearson R	P-value	Pearson R	P-value	Pearson R	P-value	Pearson R	P-value
**1**	Glycolysis and Gluconeogenesis	0.346	5.706E-15	0.420	1.637E-27	0.402	9.618E-13	0.448	2.213E-07	0.539	1.445E-17	0.475	5.198E-16
**2**	Carbonic acid metabolism	0.271	1.538E-09	0.334	2.242E-17	0.380	2.090E-11	0.418	1.705E-06	0.519	3.134E-16	0.505	3.910E-18
**3**	Propanoate metabolism	0.283	2.456E-10	0.251	3.316E-10	0.258	8.226E-06	0.380	1.571E-05	0.462	9.126E-13	0.249	5.046E-05
**4**	Transport (golgi apparatus)	0.190	2.643E-05	0.108	7.801E-03	0.222	1.342E-04	0.262	3.498E-03	0.210	1.952E-03	0.197	1.453E-03
**5**	Purine metabolism	0.257	9.821E-09	0.247	6.039E-10	0.275	1.865E-06	0.322	2.985E-04	0.194	4.219E-03	0.179	3.801E-03
**6**	Pterin biosynthesis	0.259	8.434E-09	0.273	6.463E-12	0.155	8.139E-03	0.237	8.546E-03	0.187	6.023E-03	0.193	1.844E-03
**7**	Fatty acid metabolism	0.221	9.340E-07	0.256	1.254E-10	0.289	5.251E-07	0.422	1.285E-06	0.186	6.129E-03	0.220	3.548E-04
**8**	Histone methylation	-0.111	1.454E-02	-0.100	1.384E-02	-0.176	2.662E-03	-0.244	6.736E-03	-0.167	1.421E-02	-0.149	1.671E-02

Supplementary Table 3. Metabolites significantly correlated with hypoxia score across the metabolic clusters.

Metabolic Cluster	Biochemical	Metabolomics Cohort	
		Pearson R	P-value
M1	1-arachidonoylglycerophosphoethanolamine*	0.315	0.014132212
	1-linoleoylglycerophosphocholine	0.312	0.01530133
	1-methylimidazoleacetate	-0.288	0.025820472
	1-myristoylglycerophosphocholine	0.303	0.018679284
	1-oleoylglycerophosphocholine	0.305	0.017636277
	1-oleoylglycerophosphoethanolamine	0.256	0.048324884
	1-palmitoylglycerol (1-monopalmitin)	0.339	0.008046597
	1-palmitoylglycerophosphocholine	0.296	0.021472127
	1-stearoylglycerol (1-monostearin)	0.439	0.000446959
	1-stearoylglycerophosphocholine	0.291	0.024264484
	1-stearoylglycerophosphoethanolamine	0.310	0.01593769
	1-stearoylglycerophosphoinositol	0.330	0.01008638
	10-heptadecenoate (17:1n7)	0.476	0.000121836
	10-nonadecenoate (19:1n9)	0.496	5.58516E-05
	2'-deoxyinosine	0.282	0.029011158
	2-aminoadipate	-0.321	0.012438185
	2-aminobutyrate	0.291	0.024325184
	2-arachidonoylglycerophosphocholine*	0.398	0.001652132
	2-arachidonoylglycerophosphoethanolamine*	0.381	0.002669465
	2-docosahexaenoylglycerophosphoethanolamine*	0.413	0.001048349
	2-docosapentaenoylglycerophosphoethanolamine*	0.383	0.002535693
	2-linoleoylglycerophosphocholine*	0.294	0.022445272
	2-methylbutyroylcarnitine	0.276	0.03274471
	2-myristoylglycerophosphocholine*	0.414	0.001004668
	2-oleoylglycerophosphocholine*	0.409	0.001167302
	2-palmitoleoylglycerophosphocholine*	0.419	0.000853624
	2-palmitoylglycerol (2-monopalmitin)	0.366	0.004073035
	2-palmitoylglycerophosphocholine*	0.412	0.001069922
	4-hydroxybutyrate (GHB)	0.365	0.004127158
	5-oxoproline	0.376	0.003044098
	5,6-dihydrouracil	0.267	0.039360953
	adenine	0.303	0.018789732
	adenosine 2'-monophosphate (2'-AMP)	0.362	0.004424781
	adenosine 3'-monophosphate (3'-AMP)	0.300	0.019849418
	adenosine 5'-monophosphate (AMP)	0.391	0.001981933
	adrenate (22:4n6)	0.535	1.05219E-05
	alanine	0.300	0.020066324
	arachidonate (20:4n6)	0.460	0.000216871

aspartate	0.392	0.00193988
aspartylphenylalanine	0.316	0.013801149
beta-alanine	0.308	0.016790702
betaine	0.400	0.001564238
C-glycosyltryptophan*	0.353	0.005625445
cholesterol	0.430	0.000599993
choline	0.311	0.015586443
cis-vaccenate (18:1n7)	0.341	0.007686821
conjugated linoleate (18:2n7; 9Z,11E)	0.303	0.018689445
creatine	0.317	0.013586957
cystathionine	0.282	0.028834715
cysteine	0.318	0.013247605
cystine	0.416	0.000959009
cytidine 5'-monophosphate (5'-CMP)	0.376	0.003103401
dihomo-linoleate (20:2n6)	0.526	1.59711E-05
dihomo-linolenate (20:3n3 or n6)	0.508	3.35263E-05
dimethylarginine (SDMA + ADMA)	0.442	0.000410356
docosadienoate (22:2n6)	0.500	4.75053E-05
docosahexaenoate (DHA; 22:6n3)	0.526	1.59824E-05
docosapentaenoate (n3 DPA; 22:5n3)	0.548	5.90085E-06
docosatrienoate (22:3n3)	0.259	0.04582906
eicosapentaenoate (EPA; 20:5n3)	0.377	0.002975923
eicosenoate (20:1n9 or 11)	0.481	9.9709E-05
epiandrosterone sulfate	-0.315	0.014320573
ethanolamine	0.383	0.002550808
flavin adenine dinucleotide (FAD)	0.393	0.001905679
fructose	0.282	0.028972493
fructose-6-phosphate	0.444	0.000381532
fucose	0.260	0.044670938
fumarate	0.386	0.002342762
gamma-glutamylalanine	0.355	0.005321372
gamma-glutamylisoleucine*	0.480	0.000104683
gamma-glutamylleucine	0.533	1.15249E-05
gamma-glutamyltyrosine	0.410	0.001125871
glucose-6-phosphate (G6P)	0.429	0.000625528
glutamate	0.440	0.000438605
glycerol	0.434	0.000527153
glycerol 2-phosphate	0.372	0.003390911
glycerol 3-phosphate (G3P)	0.271	0.036523081
glycerophosphorylcholine (GPC)	0.267	0.039331921
glycine	0.417	0.000910666
glycochenodeoxycholate	-0.317	0.013635432

glycocholate	-0.382	0.002600885
glycylglycine	0.315	0.014122248
glycylleucine	0.261	0.044241095
glycylvaline	0.386	0.002342992
guanine	0.499	4.97768E-05
guanosine	0.414	0.000995648
hydroxyisovaleroyl carnitine	0.341	0.007648693
hypoxanthine	0.473	0.000136616
inositol 1-phosphate (I1P)	0.448	0.000326781
Isobar: fructose 1,6-diphosphate, glucose 1,6-diphosphate	0.303	0.018491085
isobutyrylcarnitine	0.300	0.019829179
kynurenine	0.299	0.020325325
lactate	0.405	0.001332822
laurate (12:0)	0.285	0.02723575
leucine	0.422	0.000773596
linoleate (18:2n6)	0.445	0.000364198
linolenate [alpha or gamma; (18:3n3 or 6)]	0.429	0.000635433
lysine	0.321	0.012389596
malate	0.455	0.000256215
malonate (propanedioate)	0.315	0.014264657
maltose	0.264	0.04186372
mannitol	0.288	0.025453985
mannose	0.373	0.003344686
mannose-6-phosphate	0.382	0.002567641
margarate (17:0)	0.514	2.69679E-05
methionine	0.258	0.046818073
methylphosphate	0.372	0.003433121
myristate (14:0)	0.428	0.000644344
myristoleate (14:1n5)	0.460	0.000214517
N-acetylalanine	0.422	0.000779089
N-acetylglucosamine	0.292	0.023607491
N-acetylglucosamine 6-phosphate	0.472	0.000141957
N-acetylmethionine	0.357	0.005066421
N-acetylserine	0.424	0.000743356
N-acetylthreonine	0.275	0.033486048
N1-methyladenosine	0.377	0.002989946
N1-methylguanosine	0.378	0.002893176
N6-acetyllysine	0.342	0.007520521
nicotinamide	0.348	0.006374524
octanoylcarnitine	-0.265	0.040751983
oleate (18:1n9)	0.448	0.000326946
ornithine	0.290	0.024784327

	palmitate (16:0)	0.459	0.000223488
	palmitoleate (16:1n7)	0.473	0.000133094
	phenylalanine	0.268	0.038440756
	phosphate	0.427	0.000665245
	phosphoethanolamine	0.355	0.005403752
	pipecolate	0.376	0.003058409
	proline	0.435	0.000522514
	putrescine	0.288	0.025407587
	quinolinate	0.264	0.041827917
	riboflavin (Vitamin B2)	0.394	0.0018239
	ribose	0.355	0.005365747
	ribulose	0.272	0.035332115
	S-adenosylhomocysteine (SAH)	0.315	0.014114082
	S-adenosylmethionine (SAM)	0.328	0.010446586
	sedoheptulose-7-phosphate	0.269	0.037487739
	serine	0.397	0.001678609
	sorbitol	0.390	0.002063087
	sphinganine	0.256	0.048387798
	stearate (18:0)	0.492	6.50947E-05
	stearidonate (18:4n3)	0.337	0.008370397
	taurochenodeoxycholate	-0.310	0.01579768
	threonine	0.302	0.018960867
	tryptophan	0.436	0.000489797
	uracil	0.408	0.001217479
	uridine	0.346	0.006787246
	valine	0.298	0.020860067
	xylitol	0.300	0.019852023
**M2**	2'-deoxyguanosine	0.752	0.012058245
	5-oxoproline	0.700	0.024341203
	alpha-ketoglutarate	-0.634	0.04921449
	creatinine	-0.721	0.018670404
	cystine	0.635	0.048590293
	gamma-glutamylisoleucine*	0.667	0.035294331
	gamma-glutamylleucine	0.706	0.02248533
	glucose	-0.671	0.033761036
	methylphosphate	0.715	0.020067168
	ophthalmate	-0.683	0.029630474
	thymine	0.699	0.024597734
**M3**	1-oleoylglycerophosphoethanolamine	-0.616	0.00227501
	2'-deoxyguanosine	-0.544	0.008916849
	2-hydroxyglutarate	-0.660	0.000840832
	3-aminoisobutyrate	-0.444	0.038680584

	erythronate*	-0.424	0.049029984
	glycocholate	-0.679	0.000507113
	ribitol	-0.437	0.041771556
	scyllo-inositol	-0.603	0.002991535
	thymine	-0.479	0.024191357
	xanthine	-0.472	0.026516299
